# Periodic Fever, Aphthous Stomatitis, Pharyngitis, and Cervical Adenitis Syndrome: A Rare Etiology of Fever in Adults

**DOI:** 10.7759/cureus.14749

**Published:** 2021-04-29

**Authors:** Sérgio Ferreira Cristina, Adriana Costa, Manuel Toscano, Estela Kakoo Brioso, Patrícia Cipriano

**Affiliations:** 1 Internal Medicine, Hospital De Cascais Dr. José De Almeida, Lisbon, PRT; 2 Pediatrics, Hospital Prof. Doutor Fernando Fonseca, Lisbon, PRT; 3 Pediatrics, Hospital De Cascais Dr. José De Almeida, Lisbon, PRT

**Keywords:** pfapa, periodic fever, autoinflammation, pharyngitis, immunology

## Abstract

We report the case of a 28-year-old male, with a past history of recurrent pharyngitis and tonsillectomy, who presented to the emergency department with fever, pharyngitis, and cervical adenitis. Inflammatory markers were elevated and the patient was initially started on ceftriaxone with remission after four days. However, the symptoms recurred three weeks later and an autoinflammatory disease was suspected. After exclusion of other illnesses, a diagnosis of periodic fever, aphthous stomatitis, pharyngitis, and cervical adenitis (PFAPA) syndrome was confirmed. The patient was successfully treated with a single dose of 60 mg of prednisolone at the beginning of the flare.

PFAPA syndrome has been classically diagnosed solely in children but cases in adults are being increasingly recognized. Despite the increasing evidence of the delayed onset of PFAPA syndrome during adulthood, no specific tools are available to detect it and diagnosis is currently based on clinical diagnostic criteria, which have very low specificity and are tailored to pediatric patients. This case report stresses the need to consider this entity seriously despite its rarity, even among the adult population, so as to reduce iatrogenesis, start appropriate therapy in a prompt manner, and improve the quality of life of PFAPA patients.

## Introduction

Periodic fever, aphthous stomatitis, pharyngitis, and cervical adenitis (PFAPA) syndrome is the most frequent non-hereditary autoinflammatory disorder in childhood, with 90% of cases occurring before the age of five, though reports of its appearance in adults are on the rise [1,2]. There is a slight male predominance (55-71%) and no predilection for a particular ethnic or racial group [3,4].

In this report, we present the case of a 28-year-old male with PFAPA syndrome who was successfully treated with a single dose of 60 mg of prednisolone at the beginning of the flare.

## Case presentation

History and physical exam

A 28-year-old male presented to the general emergency department with a fever (maximum temperature of 38.8 °C) that had started two months ago. The fever was episodic, with each episode usually lasting four days and recurring every three weeks. These episodes were always accompanied by odynophagia that subsided when the fever disappeared, and occasionally by painful oral aphthous ulcers that tended to last longer than the febrile episode. He had been seen by different physicians during each of the episodes, and these consultations had invariably led to a diagnosis and treatment of upper respiratory airway infection. The patient had a previous history of multiple episodes of pharyngitis (usually treated with antibiotics) during his childhood, adolescence, and early adult life, which had ceased at the age of 24 after he had undergone elective tonsillectomy. He denied any history of recent travels, contact with animals, or unprotected sex. The physical findings upon admission were as follows: low-grade fever (38.5 °C), tachycardia (130 beats per minute), hyperemia of the oropharynx, and unilateral enlarged cervical lymph nodes.

The patient was initially diagnosed with a respiratory infection, started on empirical treatment with intravenous ceftriaxone, and was admitted to the Internal Medicine ward for investigation of a comorbid condition predisposing to recurrent upper airway infections or an alternate diagnosis. The patient completed a seven-day course of ceftriaxone and the fever was only present during the first four days. He was discharged and referred for outpatient follow-up.

Three weeks after the discharge, the patient had another episode of fever, odynophagia, and oral ulcers that remitted spontaneously after four days.

Investigations

Laboratory work at the time of admission showed elevation of inflammatory markers [leukocytosis of 17 x 10^9^ cells/L with 81.2% of neutrophils and C-reactive protein (CRP) of 9.28 mg/dL (reference range: <0.5 mg/dL)]; the chest radiograph was normal.

Infection studies were all negative throughout the hospital stay, namely blood and sputum cultures, acid-fast bacillus testing, rapid plasma reagin (RPR), and serology for Epstein-Barr virus, cytomegalovirus, human immunodeficiency virus (HIV), hepatitis B and C virus, *Coxiella burnetti*, *Rickettsia connori*, and antigens for *Legionella*, S*treptococcus pneumoniae* (*S.* *pneumoniae)*, and influenza A and B.

The peripheral blood smear showed a moderate anisocytosis and left skewing of granulocyte; the serum protein electrophoresis revealed a slightly reduced albumin concentration (3.0 g/dL) consistent with an inflammatory state, and we observed an elevation of immunoglobulin A and D [574 mg/dL (reference range: 70-400 mg/dL) and 284 kUI/L (reference range: <100 kUI/L), respectively]. Complement proteins were normal. Antinuclear antibodies were positive (titer 1:320; fine granular pattern; negative mitotic cells and nucleoli), but anti-double-stranded DNA (anti-dsDNA), anti-SSA, and anti-SSB were all negative.

**Figure 1:**
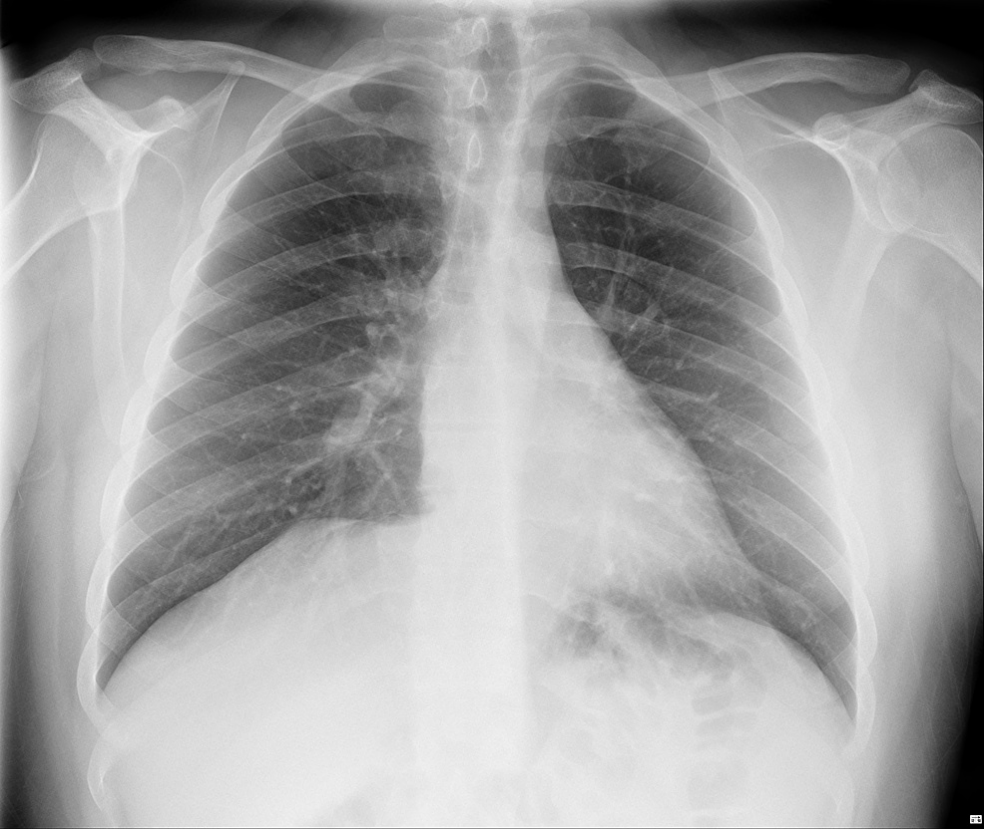
Normal chest X-ray of the patient

**Figure 2:**
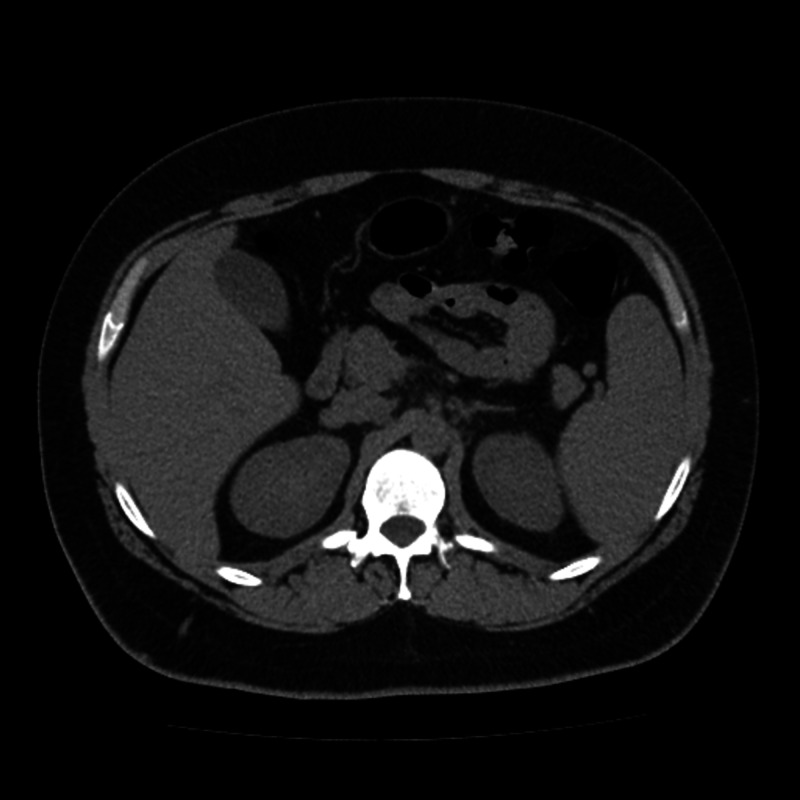
Normal abdominal CT of the patient CT: computed tomography

**Figure 3:**
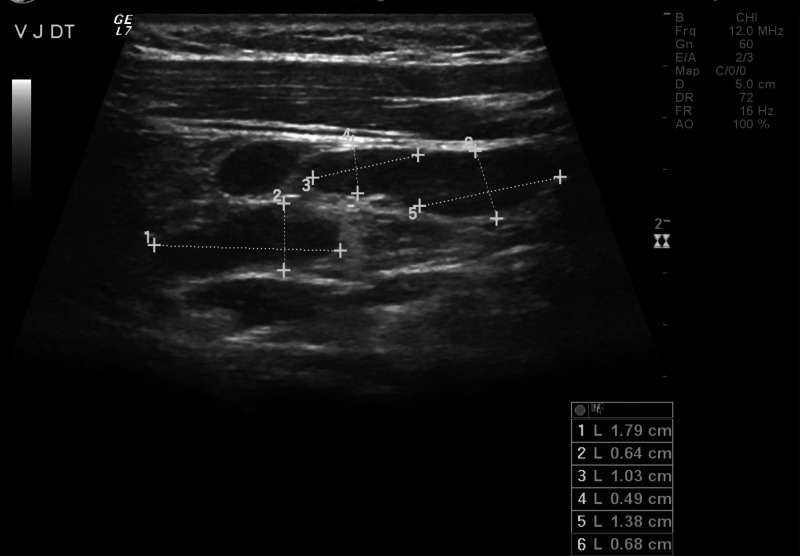
Cervical ultrasound of the patient The cervical ultrasound showed bilateral multiple lymph node conglomerates (indicated by the dotted lines with the respective dimensions on the right lower corner)

Imaging studies of the thorax (Figure 1) and abdomen (Figure 2) were normal, and a cervical ultrasound showed multiple bilateral lymph node conglomerates, with the larger one measuring 30 x 10 mm (Figure 3). Although initially considered reactive to the inflammatory process, a second ultrasound was performed after discharge when the patient was asymptomatic and, since the largest conglomerate was still present, it was aspirated using a fine needle. The cytology did not show any signs of a lymphoid neoplasm.

The inflammatory markers subsided before discharge and were completely negative during the asymptomatic period.

Differential diagnosis

In a patient presenting with recurrent fever, the differential diagnosis must encompass a vast number of etiologies, including infection, autoimmunity/autoinflammation, and neoplasia.

The presence of fever and pharyngitis and the disappearance of symptoms after the initiation of antibiotics raised the suspicion of recurrent infection of the pharynx as the etiology for this clinical picture. The elevation of inflammatory markers only during the episodes was also suggestive of an infection. However, the fixed frequency of the episodes (every three weeks), the long duration of these recurrent episodes (since childhood), and the absence of other signs of immunodeficiency (no recurrent infections in other locations, absence of complications like abscesses, normal spleen on abdominal imaging, negative serology for HIV, and no reduction in immunoglobulins levels) reduced the likelihood of an infection causing this presentation. It seems far more likely that the clinical improvement with antibiotics resulted from a self-limiting process, as was observed when the subsequent episode was not treated with medications.

Hematologic malignancies, particularly the ones derived from the lymphoid lineage, should always be considered in a patient presenting with recurrent fever and lymph node enlargement. In our patient, there were accompanying symptoms (odynophagia and aphthous ulcers) besides the cervical adenopathy, which suggested a different etiology. Also, the fact that these episodes were of several years' duration and the absence of constitutional symptoms like weight loss, night sweats, or worsening fatigue made this diagnosis less likely. Nonetheless, the patient was submitted to a fine needle aspiration of the adenopathy, which showed no signs of malignancy.

The long history of febrile episodes, associated with a periodic recurrence every three weeks, strongly suggested an autoinflammatory syndrome, especially because recurrent fever is the hallmark of autoinflammation. Although frequently diagnosed during childhood, some patients only start to show symptoms later in life or when they reach adulthood with no diagnosis despite being symptomatic. Febrile episodes in familial Mediterranean fever and tumor necrosis factor receptor-associated periodic syndrome (TRAPS) are usually accompanied by abdominal pain, arthralgia, conjunctivitis, myalgia, or rash, and cryopyrin-associated periodic syndromes (CAPS) episodes that are usually present since birth (generally associated with rashes and sometimes triggered by cold exposure). Our patient did not present with any of these characteristics and so we ruled these diagnoses out. The presence of cervical adenopathy and elevation in immunoglobulin A and D was compatible with hyperimmunoglobulin D syndrome but, similar to the previously described syndromes, hyperimmunoglobulin D syndrome is frequently associated with pain episodes, diarrhea, vomiting, lymphadenopathy outside the cervical area, and arthralgia, which the patient did not have. Additionally, none of these autoinflammatory syndromes are truly periodic, with inter-attack periods varying in the same patient. Our patient presented with a complaint of true periodic fever (three weeks' intervals between episodes), which is only suggestive of PFAPA and cyclic neutropenia (which is not an autoinflammatory syndrome and is characterized by neutropenia during febrile episodes, a feature not present in our patient). The presence of periodic fever accompanied by aphthous stomatitis, pharyngitis, and adenitis was strongly suggestive of PFAPA syndrome. The resolution of symptoms following tonsillectomy was also characteristic of this entity and, although infrequent, there are several case reports of recurrence after remission with tonsillectomy. To confirm the diagnosis of PFAPA, we administered 60 mg of prednisolone orally at the onset of the episodes, which resulted in complete resolution of symptoms in hours with no recurrence of fever for another three weeks. This finding is highly specific to PFAPA among patients presenting with features of an autoinflammatory syndrome and hence the diagnosis was confirmed.

Treatment and outcome

Treatment options were discussed with our patient and we agreed upon episodic treatment with prednisolone 60 mg.

One year after the diagnosis, the patient continues to have episodic attacks at three-week intervals, which he has been controlling with single-dose prednisolone. No other clinically significant symptoms were noted throughout the follow-up period.

## Discussion

The pathogenesis of this syndrome is not completely understood, but a multifactorial origin, probably based on a polygenic pattern of susceptibility, is the most probable rational hypothesis [5]. Cellular responses associated with this condition are generally distinct from those seen in infections. Inflammation is derived from activation of the innate immune system, but T-cell dysfunction is also present in PFAPA patients. Monocytes are increased, and eosinophils and lymphocytes are often decreased during febrile episodes. Platelets are increased in the afebrile intervals [6,7]. Periodic fever is the hallmark of the episodes of PFAPA syndrome. Flares usually last three to seven days and recur at intervals of two to eight weeks, resulting in highly predictable episodes in most pediatric cases [1]. Fever begins abruptly, often accompanied by chills. Prodromal symptoms of malaise, irritability, sore throat, and aphthous ulcers may occur during the preceding day. Aphthous ulcers, usually on the lips or buccal mucosa, occur in approximately 40-70% of patients. Pharyngitis with tonsillar exudates and cervical adenopathy accompany the fever [2]. Symptoms such as headache, myalgia, and abdominal pain are common but not specific to PFAPA syndrome [8].

Clinical evaluation remains the mainstay of diagnosis of PFAPA, rather than laboratory testing. Biomarkers during the febrile state, such as elevated CRP in the absence of elevated procalcitonin, vitamin D, cluster of differentiation 64 (CD64), C-X-C motif chemokine 10 (CXCL10), erythrocyte sedimentation rate, serum amyloid A, tumor necrosis factor α (TNF-α), interleukin 1β (IL-1β), and IL-6, mean corpuscular volume, and other nonspecific inflammatory mediators, may aid in the diagnosis in the absence of an infectious explanation for the fever [2,9]. Among the mentioned biomarkers, only CD64 and CXCL10 are uniquely elevated in PFAPA and not in other periodic fever syndromes or infectious conditions [2].

The diagnosis is currently based on the modified Marshall’s criteria proposed in 1999, which requires the presence of regularly recurring fevers with an early age of onset (<5 years of age), constitutional symptoms in the absence of upper respiratory infection (with at least one of the following clinical signs: aphthous stomatitis, cervical lymphadenitis, or pharyngitis), exclusion of cyclic neutropenia, completely asymptomatic intervals between episodes, and normal growth and development for PFAPA to be classified [3]. These criteria have good sensitivity but lack specificity [3]. Hence, their power remains limited. There are several ongoing investigations aiming to establish new criteria [1,10,11].

The PFAPA syndrome has been classically diagnosed solely in children aged less than five years of age, but there has been increasing evidence that suggests a wider expression pattern of the disease, with the emergence of cases in older children and adults with no gender predominance. Nevertheless, PFAPA syndrome is still recognized more frequently in children, and hence the diagnostic delay is strikingly higher in adults. Data describing the exact incidence of delayed onset PFAPA syndrome are still scarce [12].

It has been postulated that, similar to children, who present some form of delayed maturation of the immune system (and generally improve once the capacity of the immune system is regained in adulthood), adults with PFAPA syndrome are individuals whose immune system never reached maturation and are therefore prone to stimuli triggering PFAPA episodes.

A comprehensive comparison between pediatric and adult patients suggests that the frequency of flares is significantly higher in children but the duration of the fever episode is significantly longer in adults [13]. Clockwork periodicity of fever and recurrent pharyngitis is more frequently observed in childhood, but no differences were identified for aphtosis and cervical lymphadenopathy [13]. Arthralgia, fatigue, myalgia, headache, rashes, thoracic or periorbital pain, and conjunctivitis are also more common in adult PFAPA patients, which might make the setting of adult-onset PFAPA syndrome more symptom-heavy [12].

Despite the increasing evidence of the delayed onset of PFAPA syndrome during adulthood, no specific diagnostic tools are available and diagnosis is currently based on clinical diagnostic criteria, which have very low specificity and are tailored to pediatric patients (application in adults requires ignoring the first and fifth items). Cantarini et al. have recently proposed a set of diagnostic criteria for adult-onset PFAPA, which include fever episodes associated with cervical lymphadenitis, fever attacks associated with erythematous pharyngitis, increased inflammatory markers during fever attacks, and the lack of clinical and laboratory signs of inflammation between flares [14]. The occurrence of recurrent fever with erythematous pharyngitis appears to be the symptom most strongly associated with a diagnosis of PFAPA syndrome in adulthood. The simultaneous occurrence of two or three cardinal PFAPA signs does not appear to show any statistical difference, while the occurrence of only one cardinal manifestation is more frequent in adults. Since adult presentations are characterized by a wider repertoire of inflammatory signs, the onset in adulthood might leave the disease misdiagnosed. Hence, PFAPA should be considered in any adult with recurrent unexplained episodes of fever.

Treatment of PFAPA in children includes pharmacological and surgical options, but an optimal treatment method is not yet established. Given the favorable natural history, treatment is optional, and the risks of symptomatic treatment must be weighed against the risk of adverse effects. Nonsteroidal anti-inflammatory agents and antipyretics have shown poor results in resolving the symptoms of PFAPA syndrome. However, glucocorticoids are highly effective in aborting the attacks. A single dose of prednisone (1-2 mg/kg) or betamethasone (0.1-0.2 mg/kg) given at the onset of an episode is highly effective and reduces fever rapidly but does not prevent future PFAPA episodes from occurring [2,15].

Colchicine prophylaxis may be an interesting option for patients with frequent episodes, although complete responses have been rarely observed [16]. The medical literature contains some PFAPA reports supporting the usefulness of cimetidine prophylaxis, though evidence for its efficacy is weak [1]. IL-1 blockade has been the most recent development in the treatment for PFAPA, based on the research regarding IL-1β dysregulation in the pathogenesis of PFAPA. Drugs such as anakinra, rilonacept, and canakinumab have shortened the duration of febrile episodes or induced a complete resolution of symptoms of PFAPA [2].

Most studies support the effectiveness of tonsillectomy with or without adenoidectomy in inducing remission or decreasing symptoms in most patients with PFAPA, though attacks can recur between 0.5-10 years after the removal of the tonsils [3,4]. In adult PFAPA patients, the intermittent use of a single oral corticosteroid dose is the preferred first-line treatment as it is safe, convenient, and cost-effective, while the evidence seems to suggest that tonsillectomy is less useful in adults than in children [4].

Although the first case of adult PFAPA was reported in 2006, there has been a paucity of case reports and case series since then [17]. In 2008, the first series of patients with adult PFAPA was published and raised awareness about the need for considering this entity in adults with periodic fever [4]. Similarly, in 2011, Colloto et al. reported the first case of a PFAPA diagnosis in an adult patient with a previous tonsillectomy [18]. The first description of recurrence in adults with a previous remission of PFAPA during childhood was published in 2016, and it ushered in the notion that PFAPA might not be as self-limiting as once thought [19]. To our knowledge, only one other case of adult PFAPA has been reported in Portugal [20]. The patient was a 24-year-old female who had experienced pertinent symptoms in the previous four years prior to diagnosis.

Our case differs from most published reports in adults because our patient had experienced febrile episodes since early childhood that had gone misdiagnosed and persisted till adulthood (with a brief remission period after tonsillectomy). This report highlights the need to seriously consider this entity despite its rarity, even among the adult population, to reduce iatrogenic interventions, start appropriate therapy, and thereby improve the quality of life of PFAPA patients.

## Conclusions

PFAPA syndrome should always be considered in patients reporting recurrent febrile episodes regardless of their age. Adult PFAPA patients often present with more generalized symptoms than pediatric patients, and hence a high index of suspicion is needed when dealing with this patient population. Nonetheless, in adults presenting with recurrent fever and adenopathy, it is important to exclude other more frequent and serious conditions, particularly recurrent infections and malignancies.
